# Genetic Structure of Bluefin Tuna in the Mediterranean Sea Correlates with Environmental Variables

**DOI:** 10.1371/journal.pone.0080105

**Published:** 2013-11-18

**Authors:** Giulia Riccioni, Marco Stagioni, Monica Landi, Giorgia Ferrara, Guido Barbujani, Fausto Tinti

**Affiliations:** 1 Marine Biology & Fisheries Laboratory, Department Biological, Geological & Environmental Sciences, University of Bologna, Fano (PU), Italy; 2 Laboratory of Genetics & Genomics of Marine Resources and Environment, Department Biological, Geological & Environmental Sciences, University of Bologna, Campus of Ravenna, Ravenna, Italy; 3 Department of Life Sciences and Biotechnologies, University of Ferrara, Ferrara, Italy; Institute of Biochemistry and Biology, Germany

## Abstract

**Background:**

Atlantic Bluefin Tuna (ABFT) shows complex demography and ecological variation in the Mediterranean Sea. Genetic surveys have detected significant, although weak, signals of population structuring; catch series analyses and tagging programs identified complex ABFT spatial dynamics and migration patterns. Here, we tested the hypothesis that the genetic structure of the ABFT in the Mediterranean is correlated with mean surface temperature and salinity.

**Methodology:**

We used six samples collected from Western and Central Mediterranean integrated with a new sample collected from the recently identified easternmost reproductive area of Levantine Sea. To assess population structure in the Mediterranean we used a multidisciplinary framework combining classical population genetics, spatial and Bayesian clustering methods and a multivariate approach based on factor analysis.

**Conclusions:**

F_ST_ analysis and Bayesian clustering methods detected several subpopulations in the Mediterranean, a result also supported by multivariate analyses. In addition, we identified significant correlations of genetic diversity with mean salinity and surface temperature values revealing that ABFT is genetically structured along two environmental gradients. These results suggest that a preference for some spawning habitat conditions could contribute to shape ABFT genetic structuring in the Mediterranean. However, further studies should be performed to assess to what extent ABFT spawning behaviour in the Mediterranean Sea can be affected by environmental variation.

## Introduction

Assessing the correlation between landscape features with the genetic variation of populations might lead to identifying environmental factors that are involved in the adaptive divergence of populations [1]. This issues is crucial in the marine realm for reconciling management and conservation of fishery stocks [2].

The Mediterranean is a temperate sea with sharply different oceanographic conditions in the western and eastern parts [3]. In spite of such differences, the two parts extensively exchange water masses between them and with the Atlantic. Surface (0–150 m depth) Atlantic water masses with relatively low salinity (36.2 practical salinity unit, psu) enter the Mediterranean through Gibraltar Strait [4] and move eastward along the North African coast (i.e. the Algerian current) reaching the Levantine Sea. Because evaporation is more intense in the Eastern Mediterranean Sea, salinity increases eastwards with a maximum of 38.5 psu in the Levantine Sea [5]. In the intermediate layer (150–600 m depth), the high-salinity Levantine water masses (∼39.1 psu) move westwards and outflow in the Atlantic. This circulation pattern generates anticyclonic gyres and steep gradients of temperature and salinity over short distances (e.g. the Almeria-Oran oceanographic front), which are almost permanent in the western basin and more variable in the eastern Mediterranean. In this complex and changing Mediterranean environment, the Atlantic Bluefin tuna (ABFT, *Thunnus thynnus*) shows a spatial population structure, which is stable over short and long time periods [6]–[9]. Despite its highly migratory behaviour and high potential of dispersal at the larval stages, the Mediterranean ABFT subpopulations display partially independent demographic dynamics [8]. A long-term correlation between ABFT abundance and surface temperature was revealed by time series catch analyses suggesting a potential strong influence of environmental factors on the ABFT migratory behaviour [10]. Population genetics offers powerful tools to identify connectivity and structure of marine populations, which might escape direct observation. The multivariate analysis of genetic data is particularly crucial when dealing with relatively weak genetic differences, as commonly detected in high-dispersal marine species.

Multidisciplinary seascape genetics (sensu [2]) addressed important issues in the spatial ecology of marine populations combining genetic and oceanographic data under ecological modelling [11]. The variation of seascape and environmental features may be correlated with patterns of genetic diversity in marine fish species [12], providing evidence for adaptive mechanisms [13], indeed genetic differentiation in several marine fish species [14], [15] has already been shown to correlate with seawater salinity and temperature.

Here, we test the hypothesis that dispersal and reproduction of the ABFT populations in the Mediterranean correlate with the physical environmental heterogeneity. We do that by comparing measures of ABFT genetic structure at microsatellite loci with surface temperature and salinity variation. For this purpose, we numerically and geographically expanded the ABFT sampling [9] by adding a sample collected from the easternmost spawning area known in the Mediterranean [16]. We also improved the analytical framework used in the previous analyses of ABFT population structure in the Mediterranean [6]–[8] by using a modified version of the software STRUCTURE [17], in which the basic models are extended to incorporate information on the sampling location, necessary to properly infer population structure when genetic differences between subpopulations are small. Indeed, several simulations and empirical studies showed that STRUCTURE may not accurately infer the correct number of genetic clusters in a sample when population structure is weak [18], [19]. The new method, developed by [17], groups individuals from the same sampling location, improving the performance of the analysis, but at the same time using this information only when clusters are correlated with their sampling location and allowing for the possibility that this information can be only partially, or even not at all, informative [17].

We also used the spatial Bayesian clustering models implemented in the software GENELAND [20], [21] to comparatively test the different behaviours of these two software packages in modelling the ABFT genetic structure. These models explicitly account for the spatial location of sampled observations and include a priori spatial autocorrelation in the genetic data, assuming that proximate observations tend to be more similar than distant ones. This assumption is useful for exploring possible spatial patterns that may arise when population differentiation occurs by limited gene flow influenced by the occurrence of landscape barriers. The model naturally incorporates the spatial locations of host samples for its assumption of spatial autocorrelation. The analytical approach was completed by multivariate methods that have displayed great efficiency in extracting information from genetic markers [22]–[24] because of their independence from genetic model assumptions (i.e. the Hardy–Weinberg equilibrium) and their performance to summarize the genetic variation into a few synthetic variables [25].

## Materials and Methods

### Ethics Statement

Tissue samples of the Atlantic Bluefin tuna *Thunnus thynnus* used in this study were collected from Mediterranean individuals caught during scientific research programs under the permission of the Italian Ministry of Agricultural and Forestry Policies (locations: Sardinian Traps, SAR; Adriatic Sea, ADR; Ligurian Sea, LIG) and by commercial long-liners and purse-seiners within the Total Allowed Catch quotas assigned by the International Commission for the Conservation of Atlantic Tunas (ICCAT) to National Governments (locations: Algerian coasts, ALG; Alboran Sea, ALB; Tyrrhenian Sea, STY; Cyprus coasts, CYP). No specific approval of this vertebrate work is required since the Bluefin tuna individuals sampled in this study were obtained from scientific and commercial fishing activities.

### ABFT Microsatellite Dataset

**Figure 1:**
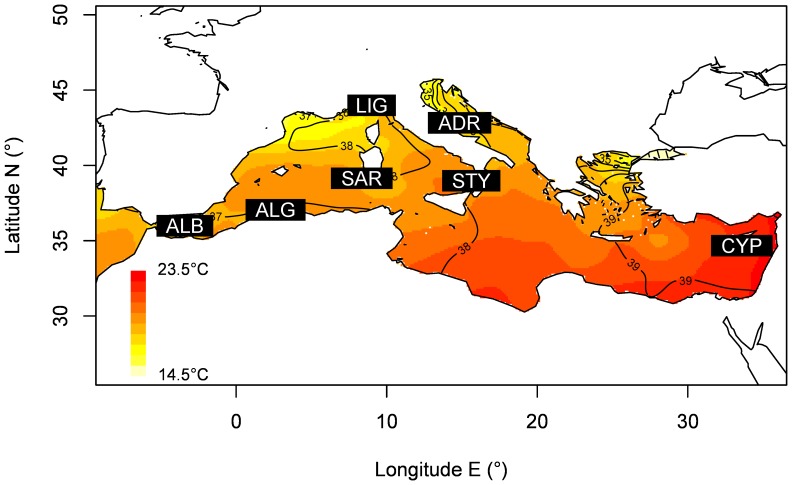
Map of surface salinity and temperature and sampling locations of *T. thynnus* in the Mediterranean. Temperature is described in colour gradient (from 14.5°C to 23.5°C) while salinity through contour map (each isoline shows a change of 1 psu). Sampling data are described in Text S1 and Tab S1(1 =  SAR, 2 =  ADR, 3 =  LIG, 4 =  ALG, 5 =  ALB, 6 =  STY, 7 =  CYP).

In this study we have reanalysed the genetic variation of six ABFT Mediterranean population samples (N = 256) previously genotyped at potentially neutral microsatellite loci by [8]. In addition, we have added to this data set a population sample collected from the Levantine Sea, off the coast of Cyprus (CYP, N = 60; Fig. 1). The CYP sample is the easternmost ABFT Mediterranean sample analysed so far for genetic variation and it was collected from an ABFT spawning area identified in the Eastern Mediterranean [16]. Therefore, the complete data set includes seven ABFT population samples (N = 316, Fig. 1) genotyped at seven microsatellite loci (Table S1, Text S1). Lab protocols and experimental conditions used for microsatellite PCR amplification and individual genotyping are reported in the SI and Dataset S1.

### Population Genetic Analyses

Allelic richness was estimated using FSTAT version 2.9.3.2 [26], expected (He) and observed (Ho) heterozygosity per locus and per sample, and the corresponding exact test for Hardy–Weinberg Equilibrium (HWE) were calculated by ARLEQUIN v. 3.5 [27] after 1,000,000 steps of Markov chains and 100,000 dememorization steps (Table S2). ARLEQUIN v. 3.5 was also used to estimate pairwise F_ST_ [28] using 10,100 permutations to obtain the null distribution of F_ST_ under the hypothesis of panmixia. A global HWE test was performed by GENEPOP 4.0 [29] using 10,000 dememorization steps of Markov chains, 20 batches and 5,000 iterations per batch. Sequential Bonferroni correction was applied for multiple test adjustment [30] (α = 0.0125).

The software STRUCTURE [31], [32] estimates Pr(X|K), the probability of the data given K genetic clusters of individuals (K = 1, 2…), by a Bayesian model-based algorithm under the HWE assumption. STRUCTURE also estimates allele frequencies in each cluster and the probability of membership of each individual to each cluster, by means of a Markov Chain Monte Carlo (MCMC) method assign genotypes to clusters minimizing the linkage disequilibrium of the clusters. The modified version STRUCTURE 2.3 developed by [17] was run allowing the use of sampling location information. This method is different from the ‘Model with prior population information’ present in the original STRUCTURE paper [31]. That model was designed to test for the presence of migrants belonging to a different location and is only useful for highly informative data, i.e. when there is strong evidence of population structure and sampling locations correspond almost exactly to the inferred clusters. An important class of Bayesian clustering models improves STRUCTURE by including information on individual geographic coordinates. We ran ten independent analyses (each with a different value of K, 1–10) using the admixture model with correlated allele frequencies [31], [32]. Each run of analysis consisted in 1,000,000 MCMC with a burn-in period of 500,000. The most likely number of clusters was inferred using both the standard method (plotting ln Pr (X|K) vs K and using the Bayes’ rule [31]) and the ΔK statistic [33] based on a rate of change in the log probability of the data. The results were averaged over multiple runs using the CLUMMP software [34] and displayed using the DISTRUCT program [35].

The spatially explicit Bayesian clustering program GENELAND 3.2.4 [20] (an extension of program R 2.12.0 [36]) was used to further investigate genetic structure. GENELAND considers individual multi-locus genotype data searching for the best fit to HWE and linkage equilibrium. GENELAND also incorporates spatial data directly under the assumption that populations are spatially organized. This program implemented different models to describe population genetic variation; we tested the correlated allele frequency models, with or without spatial information. The correlated allele frequency model accounts for the situation where some allele frequencies reflect common ancestry of different populations. The primary distinguishing factor between the spatial model and the non-spatial model in GENELAND is the assumption of spatial correlation of genotypes. Any genetic boundaries found are assumed to separate K random mating subpopulations, thus subdividing the space in a way resembling the Voronoi-Poisson tessellation [20], [37]. Ten MCMC iterations were performed, with K varying from 1 to 10, using 10,000,000 MCMC, a thinning interval of 100 generations, a maximum rate of Poisson process fixed to 316, and spatial coordinates uncertainty of 50 km. The allele frequencies prior was modelled assuming a Dirichlet distribution [38]. The assignment of individuals to subpopulations and the parameter inference were performed in a separate run as suggested by [20]. For this run, K was set to the inferred number of subpopulations and all other parameters were similar to those runs with variable K. The posterior probability of subpopulation membership was computed for each pixel of the spatial domain (200×400 pixels), using a burn-in of 500 iterations.

### Environmental Data

Seawater salinity (S, psu) and surface temperature (t, °C) data from the sampled sites were obtained from SeaDataNet Climatologies Pan-European Infrastructure for Ocean and Marine Data Management (http://gher-diva.phys.ulg.ac.be/web-vis/), a Pan-European infrastructure for managing, indexing and providing access to ocean and marine data sets and data products, acquired via research cruises and other observational activities, in situ and remote sensing. Temperature data were averaged over the period 1985–2007, while S data were averaged over the period 1900–2009.

### Multivariate Analysis

Two different ordination methods, the Correspondence Analysis (CA; [25], [39]) and the Canonical/constrained Correspondence analysis (CCA; [40]), were used to further investigate the spatial pattern of genetic variability among tuna samples. CA analysis was performed using the *R* package ADE4 1.4 [41] and ADEGENET 2.7 [42]. The CA is an ‘ordination in reduced space’ method, and it can be used to analyse tables of allele counts (Text S1). This method optimizes the χ^2^ distances among observations and therefore it can give a stronger weight to a population possessing a rare allele. As a consequence, to minimize analysis artefacts, alleles present in single copy in only one population were removed [25].

The relationship of genetic diversity with environmental factors was analysed using Canonical/constrained Correspondence analysis (CCA; see Text S1 for the method description). The contribution of each variable was assessed through correlations between environmental variables (mean-S and mean-t) and the CCA axes. This function is based on [40] algorithm and implemented in the VEGAN package [43]. In the ordination plot of CCA analysis, the constraining variables are represented by arrows directed towards the maximum change of the variable across the diagram and their lengths are proportional to the rate of change in this direction.

Multilocus genotype data were finally analysed under a model of isolation by distance (IBD). A matrix of geographical distances was obtained considering the shortest sea-paths between each pair of sampling sites using Google Earth version 6.0.2 OOB; genetic distances between populations were expressed by the ratio F_ST_/(1−F_ST_) [44]. Two additional matrices were calculated describing salinity and temperature differences between sites. The correlation between distance matrices was tested by Mantel tests [45] using the VEGAN package for R [43], and 10,000 permutations. Moreover two different types of partial Mantel test [46] allowed us to estimate partial correlation coefficients, namely between genetic and geographic distances, holding the environmental effects constant, and between genetic and environmental distances, holding the effects of geography constant.

## Results

### Bayesian Clustering Analysis

Genetic diversity in the CYP population was not significantly different from that estimated in the other Mediterranean ABFT samples (Table S2, Table S3). All pairwise F_ST_ values were significantly greater than 0 except four (comparisons ADR-LIG, ALG-STY, CYP-STY and CYP-ALG), suggesting that different Mediterranean areas roughly correspond to distinct ABFT subpopulations. The LIG-SAR F_ST_ became insignificant after the sequential Bonferroni correction (Table 1).

**Table 1:** Pairwise F_ST_s (below the diagonal) and associated P-values (above the diagonal) among *Thunnus thynnus* samples.

	ADR	STY	LIG	SAR	ALG	ALB	CYP
ADR		0.000	0.068	0.008	0.000	0.000	0.000
STY	0.016		0.000	0.001	0.126	0.001	0.595
LIG	0.006	0.021		**0.048**	0.001	0.000	0.000
SAR	0.013	0.017	0.011		0.001	0.000	0.000
ALG	0.018	0.004	0.019	0.025		0.001	0.329
ALB	0.018	0.012	0.022	0.025	0.015		0.003
CYP	0.017	−0.001	0.020	0.023	0.001	0.011	

Significance was obtained on 10,100 permutations. In bold is the value that looses significance after the Bonferroni sequential correction (α = 0.0125).

**Figure 2:**
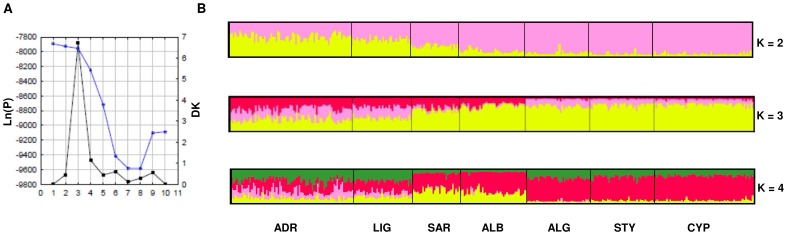
STRUCTURE analysis. A) Plot of the Log posterior probability vs K (blue line) and Evanno’s method (black line). B) Bar plot of the posterior probability of the coefficient of membership. Each vertical line represents an individual and colours represent the inferred ancestry from K ancestral populations. Results for K = 2–4 are shown.

The Bayesian analysis carried out using STRUCTURE 2.3 and the sampling location information as prior revealed the highest ΔK value for K = 3 (Evanno’s method [33], based on a rate of change in the log probability of the data), while the standard method to detect population clusters and the Bayes’ rule method [31] detected the highest probability for K = 1 (Fig. 2, a). The bar plots for K = 2–4 revealed that K = 2 could be the most plausible results, while in those for K = 3–4 most individuals showed an apparent pattern of admixture of 2 or 3 gene pools representing a clear signal of cluster overestimation. With K = 2, almost all the individuals belonging to the ALG, STY, CYP and ALB samples showed a proportion of the membership coefficients higher than 0.7 that allowed to assign these individuals to the same cluster. Conversely individuals in the SAR sample showed lower membership coefficient (0.68) and it could be grouped with ALG, STY, CYP and ALB individuals only with low statistical confidence. Similarly it was not possible to assign members of the ADR sample to one cluster with confidence, although most individuals showed values of the membership coefficients equal or higher than 0.60 for the second cluster detected by STRUCTURE. By contrast, the individuals of the LIG sample showed relatively low membership coefficients (0.48–0.52) and it was not possible to confidently assign them to either cluster.

**Figure 3:**
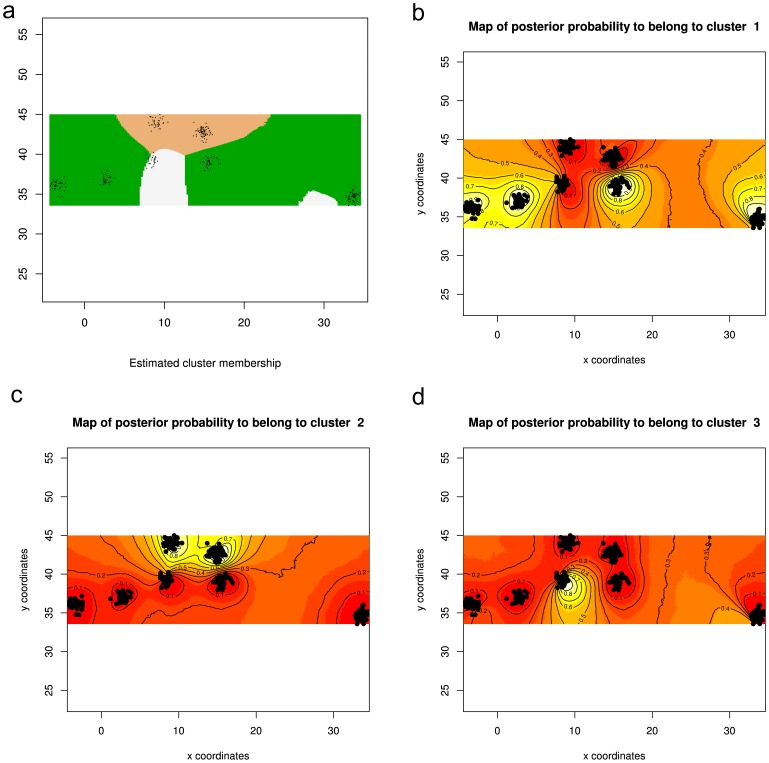
GENELAND results for K = 3 using the spatial model with correlated allele frequencies. A) Map of estimated posterior probability of population membership (by posterior mode); B-D) plots representing the assignment of pixels to the southern (B), northern (C) and central cluster (D). The highest membership values are in light yellow and the contour lines indicate the spatial position of genetic discontinuities between populations.

The spatially implicit model (with correlated allele frequencies) implemented in GENELAND did not reach convergence and this poor MCMC mixing could be due to departures from model assumptions (see GENELAND manual). On the contrary the spatially explicit model with correlated allele frequencies reached the convergence of MCMC and the most likely value of K identified was for K = 3. Individual assignments performed in GENELAND with K fixed to 3 identified distinct subpopulations splitting the southernmost samples (ALB, ALG, and CYP) and STY from the central (SAR) and northernmost samples (ADR, LIG) (Fig. 3). These findings mirrored STRUCTURE results corroborating the genetic subdivision of the southern samples and STY.

### Multivariate Analysis

**Figure 4:**
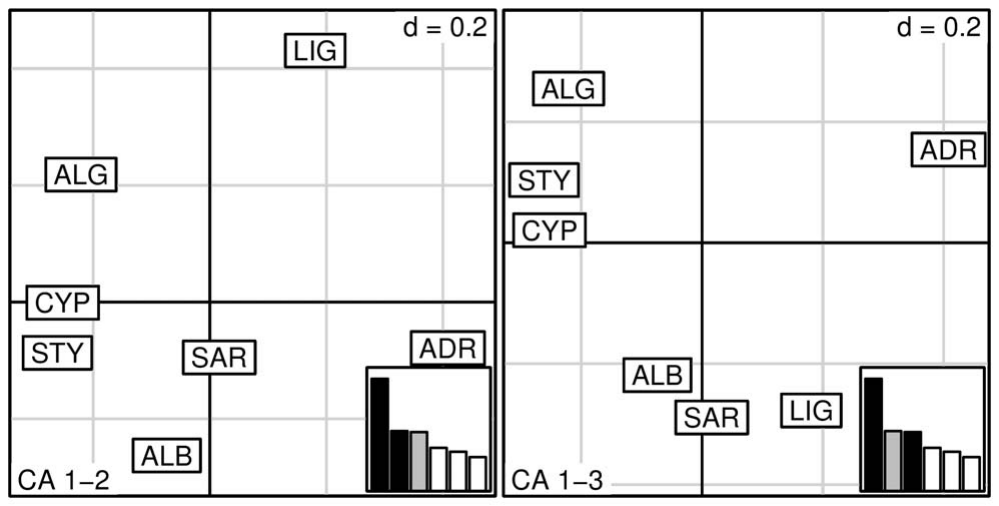
Correspondence Analysis plot of ABFT samples performed on population allele counts. The eigenvalues barplots are drawn in the bottom right corner and black bars correspond to the two axes used in the biplot (on the left CA axis 1 vs 2, on the right CA axis 1 vs 3). Grey bars represent the axes considered in the analysis but not used to draw the graph.

The Correspondence Analysis (CA) displayed a spatial pattern of differentiation largely consistent with those detected by the Bayesian clustering analysis. The CA separated the Central Northern Mediterranean ADR and LIG samples form the other samples, identified the group SAR-ALB and revealed the genetic similarity between STY and CYP (Fig. 4, Table S4).

A significant correlation between genetic distances and both geographical and mean-S distances, was revealed by the Mantel test (Table 2). On the contrary, insignificant correlations were obtained with mean-t distances. Nevertheless significant Mantel tests showed a negative correlation with both geographical and salinity distances (Table 2) and this result is not easy to explain. Therefore we further explored these correlations through partial Mantel test and Canonical/constrained Correspondence Analysis (CCA) analysis. We observed only an insignificant correlation between geographical and genetic distances (with environmental distances kept constant), while the same test carried out controlling for geographical distance produced significant results with both mean-S and mean-t distances (Table 2). Therefore, the environmental parameters appear to be reflected in the genetic population structure significantly more than geography.

**Table 2:** Results of the Mantel and partial-Mantel tests.

association	Mantel R	P-value
D∼D_g_	−0.2221	<0.001
D∼D_S_	−0.2299	<0.001
D∼D_t_	0.2312	NS
	**Partial R**	**P-value**
D∼D_g_ [D_S_]	0.0796	NS
D∼D_g_ [D_T_]	0.3029	NS
D∼D_S_ [D_g_]	−0.2301	<0.001
D∼D_t_ [D_g_]	−0.3817	<0.001

Matrix correlations describe the association between genetic and geographical/environmental distances. Partial Mantel correlations test for the variance in genetic distances among sites explained by geographical distance controlling for environmental factors (in square brackets) and by environmental distances (independent of geographical distance, in square brackets) respectively. D = genetic distance (F_ST_/(1−F_ST_)); D_g_ = geographical distance; D_S_ = mean-S distance; D_t_ = mean-t distance).

**Figure 5:**
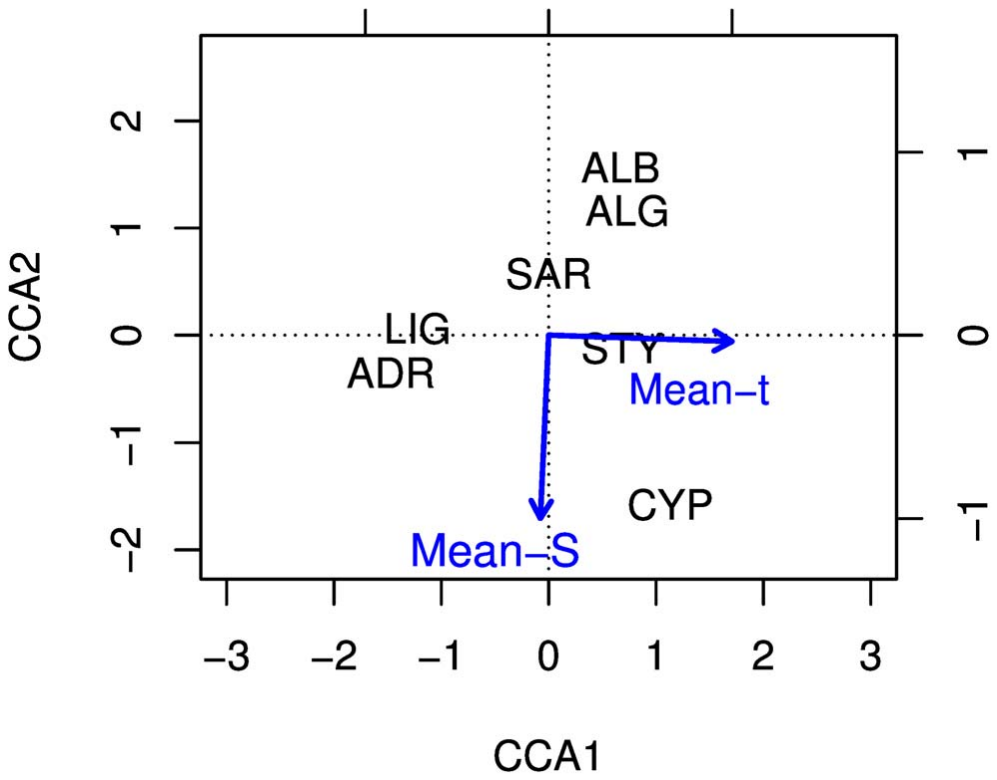
Canonical/constrained Correspondence Analysis ordination plot of ABFT samples. The environmental variables are represented by arrows: Mean-S = salinity; Mean-t = temperature.

The CCA analysis was carried out using mean-S and mean-t data as constraining factors and correlations at both axes were significant at the ANOVA 95%-significance test (CCA1 = 0.04, CCA2 = 0.05, Tables S4, S5). The CCA biplots clearly indicated the presence of a strong relationship of the mean-S with CYP and of the mean-t with both STY and CYP samples, showing a correlation of CYP sample with the highest values of salinity and temperature (Fig. 5).

The CCA analysis contributed to highlight that ABFT seems genetically structured along two environmental gradients: a longitudinal, West-to-East gradient associated to the variation of temperature and a latitudinal, North-to-South gradient associated to the variation of salinity. Moreover this analysis confirmed the clustering results obtained using GENELAND, separating the northern (ADR, LIG), from the central-southern samples (ALG, ALB, STY, CYP) and differentiating the SAR sample from all the others (Fig. 5).

## Discussion

In this study we detected the presence of at least two ABFT genetically differentiated subpopulations in the Mediterranean and we showed that this genetic structure appears to be correlated with both seawater salinity and surface temperature variation. Previous work [6]–[8] has consistently shown signals of genetic structuring in the Mediterranean ABFT populations through the detection of significant F_ST_s. Nevertheless the level of genetic divergence detected by [8] (F_ST_s’ range 0.011–0.021) was higher than in previous surveys [6], [7] and it was also supported by evidence of significant genetic differentiation between historical population samples (F_ST_ = 0.02) showing that such structuring of the Mediterranean ABFT is probably stable through time. These results, along with the evidence for a recent colonization of the Mediterranean (<20 Kya, [47]), suggested that factors other than genetic drift could produce these levels of genetic differentiation.

An important improvement of this study with respect to previous work focussing on the ABFT population genetic structure [6]–[8], is the inclusion for the first time of a sample from the eastern part of the Mediterranean (CYP, Levantine Sea), thus extending the analysis beyond the Adriatic [8] and Ionian [6], [7] Seas. The Bayesian analysis carried out using the new version of STRUCTURE program ([17]; Fig. 2b) provided an unforeseen picture clustering the CYP sample with the southern samples from the Western and Central Mediterranean (ALB, ALG and STY) that instead were separated from the northernmost samples of the same areas (ADR and LIG). This latitudinal, south-to-north sub-structuring (also observed in several other marine organisms [15], [48]–[52]) suggested a more complex pattern of genetic differentiation of ABFT in the Mediterranean than the previously detected longitudinal, west-to-east separation [6], [7], [9], [53]. However the results from STRUCTURE analysis showed that population differences are indeed minor, and did not allow us to unequivocally choose between the existence of one or three genetic clusters using classical methods for the estimation of K. Nevertheless, a deeper inspection of barplots for K = 1–4 and of membership coefficients of each sample permitted to identify K = 2 as a reliable solution reconciling F_ST_s and Bayesian results. The GENELAND method identified the spatial explicit model with correlated allele frequencies as the most reliable after MCMC convergence analysis, for this reason it was considered to be the most suitable to describe the genetic structure of Mediterranean ABFT. Considering this model to describe the genetic diversity of samples, GENELAND detected for K = 3 the highest posterior probability and further inspection of the results allowed us to rule out the presence of ‘ghost’ clusters (Fig. 3). These three clusters, separating the northern samples from the southern, remind somewhat the pattern identified by STRUCTURE for K = 2, although GENELAND was able to refine this result clearly separating the SAR sample from the northern and southern samples (Fig. 3). Multivariate analysis corroborated these findings but did not rely on assumption about the underlying population model.

In the last ten years the improved methods of satellite tagging provided insights into tuna movements [54], confirming the transatlantic migration of this pelagic fish and highlighting only limited individual movements in this part of the Mediterranean Sea [55]. The integration of tagging [53]–[55] and genetic data [6], [7], [10] suggests the presence of some tuna sub-populations displaying different migratory behaviour. The intriguing hypothesis that at least three different ABFT sub-populations exist in the Mediterranean was formulated by [10]. Our results contributed to support this scenario by indicating that ABFT could be in fact a metapopulation characterized by complex sub-population dynamics and by partial reproductive isolation. The present work adds an important detail to the picture, namely the finding that genetic structuring correlates with environmental variables, providing further evidence that environmental conditions affect ABFT reproductive behaviour. The Mantel test revealed indeed a significant correlation of ABFT allele frequencies with salinity in the Mediterranean. We also showed that genetic distances correlate with both salinity and surface temperature when the effect of geographic distances is partialled out, whereas the correlation of genetic and geographical distances appears to be a statistical artefact, due to the correlation between geography and environmental variables. The correlation between genetic and environmental variation is a further piece of evidence about the influence of environmental factors on ABFT population dynamics. Indeed Massimo Sella at the beginning of the 20^th^ century [56], [57] already proposed that salinity and temperature influence both water regime and tuna movements. More recently, several experimental and modelling studies suggested a direct involvement of environmental factors on ABFT spatial dynamics and migration pattern. ABFT spawning behaviour [58], [59], called “repeat homing”, is a process of spatial learning of water-mass conditions optimal for spawning (e.g. SST >20.5°C with a preference form 21.5 to 26.5°C in the Western Mediterranean; [60]). Because ABFT larval occurrences were associated with the confluence of inflowing Atlantic waters and saltier resident surface waters [61]–[63], it has been argued that ABFT spawners preferentially target as spawning grounds mesoscale hydrographical structures whose chemical-physical and productivity conditions favour egg buoyancy and hatching as well as larval retention and survival [60], [64]–[66]. In the Western and Central Mediterranean, the constancy of large-scale surface currents spatially portioned hydrographical structures that are more stable than in the Eastern Mediterranean [67]. Moreover, the spatio-temporal variability at the regional scale and the mesoscale circulation [67], [68] form patches of high-density larvae with very limited extension (from 10 to 13 nautical miles; [60], [65], [66]) favouring ABFT genetic structuring. As in other several fish, the variation of complex environmental and oceanographic conditions (among which SST and salinity might represent two functional and operative proxies of such variation) and of life history traits, as spawners’ habitat preferences and larval phase features, can likely influence population connectivity in the Mediterranean ABFT [69]. Our findings suggested that spawning ground choice could affect ABFT genetic differentiation in the Mediterranean. The significant relationship detected between salinity, temperature and population divergence, represents only a starting point, because many other environmental factors could be involved and interact with each other producing this pattern of genetic differentiation. In particular, we revealed two genetic gradients significantly linked to the variation of chemical-physical conditions: a west-east gradient linked to salinity and a north-south gradient linked to surface temperature values, with strongest relationship of the CYP sample with areas of high salinity and temperature.

This survey showed clearly that, to refine the subtle but significant signal of genetic structure detected in highly migratory fish, it is essential to combine several biostatistical tools based on different assumptions [17], [21], [70]–[73]. Nevertheless methodological differences can be fundamental in the identification of the signal of genetic differentiation. The integration of landscape ecology with population genetics can enable the detection of environmental factors that may promote or constrain the divergence detected, providing also a refinement of the genetic structure identified through methods of analysis completely independent from any kind of genetic model or assumptions [25].

## Supporting Information

Table S1 Sampling data of the *Thunnus thynnus* samples.
(DOC)

Table S2 Summary statistics at the seven microsatellite loci of *Thunnus thynnus* samples.
(DOC)

Table S3 Gene diversity and Hardy-Weinberg equilibrium deviation test of *Thunnus thynnus* samples.
(DOC)

Table S4 Percentages of variations explained by CA and CCA axis.
(DOC)

Table S5 Ordination scores of mean temperature (Mean-t) and mean salinity (Mean-S) values.
(DOC)

Text S1 DNA extraction, Microsatellite marker analysis protocols, Correspondence Analysis (CA) and Canonical/Constrained Correspondence Analysis (CCA) results and references.
(DOC)

Dataset S1 Microsatellite dataset in gtx format.
(GTX)

## References

[1] Manel S, Schwartz MK, Luikart G, Taberlet P (2003) Landscape genetics: combining landscape ecology and population genetics. Trends in Ecology & Evolution 18: 189–197.

[2] Selkoe KA, Henzler CM, Gaines SD (2008) Seascape genetics and the spatial ecology of marine populations. Fish and Fisheries 9: 363–377.

[3] Robinson A, Leslie W, Theocharis A, Lascaratos A (2001) Mediterranean Sea circulation. Encyclopedia of Ocean Science vol 3: Academic Press, San Diego, CA 1689–1705.

[4] Millot C (1999) Circulation in the Western Mediterranean Sea. Journal of Marine Systems 20: 423–442.

[5] Patarnello T, Volckaert F, Castilho R (2007) Pillars of Hercules: is the Atlantic-Mediterranean transition a phylogeographical break? Molecular Ecology 16: 4426–4444. 17908222 10.1111/j.1365-294X.2007.03477.x

[6] Carlsson J, McDowell JR, Diaz-Jaimes P, Carlsson JE, Boles SB, et al (2004) Microsatellite and mitochondrial DNA analyses of Atlantic bluefin tuna (*Thunnus thynnus thynnus*) population structure in the Mediterranean Sea. Molecular Ecology 13: 3345–3356. 15487994 10.1111/j.1365-294X.2004.02336.x

[7] Carlsson J, McDowell JR, Carlsson JE, Graves JE (2007) Genetic identity of YOY bluefin tuna from the eastern and western Atlantic spawning areas. Journal of Heredity 98: 23–28. 17158466 10.1093/jhered/esl046

[8] Riccioni G, Landi M, Ferrara G, Milano I, Cariani A, et al (2010) Spatio-temporal population structuring and genetic diversity retention in depleted Atlantic Bluefin tuna of the Mediterranean Sea. Proceedings of the National Academy of Sciences of the United States of America 107: 2102–2107. 20080643 10.1073/pnas.0908281107 PMC2836650

[9] Boustany AM, Reeb CA, Block BA (2008) Mitochondrial DNA and electronic tracking reveal population structure of Atlantic bluefin tuna (*Thunnus thynnus*). Marine Biology 156: 13–24.

[10] Fromentin JM (2009) Lessons from the past: investigating historical data from bluefin tuna fisheries. Fish and Fisheries 10: 197–216.

[11] Gerlach G, Atema J, Kingsford M, Black K, Miller-Sims V (2007) Smelling home can prevent dispersal of reef fish larvae. Proceedings of the National Academy of Sciences of the United States of America 104: 858–863. 17213323 10.1073/pnas.0606777104 PMC1783404

[12] Knutsen H, Andre C, Jorde PE, Skogen MD, Thuroczy E, et al (2004) Transport of North Sea cod larvae into the Skagerrak coastal populations. Proceedings of the Royal Society of London Series B-Biological Sciences 271: 1337–1344. 10.1098/rspb.2004.2721 PMC1691739 15306331

[13] Funk DJ, Nosil P, Etges WJ (2006) Ecological divergence exhibits consistently positive associations with reproductive isolation across disparate taxa. Proceedings of the National Academy of Sciences of the United States of America 103: 3209–3213. 16492742 10.1073/pnas.0508653103 PMC1413886

[14] Bekkevold D, Andre C, Dahlgren TG, Clausen LAW, Torstensen E, et al (2005) Environmental correlates of population differentiation in Atlantic herring. Evolution 59: 2656–2668. 16526512

[15] Cimmaruta R, Bondanelli P, Nascetti G (2005) Genetic structure and environmental heterogeneity in the European hake (*Merluccius merluccius*). Molecular Ecology 14: 2577–2591. 15969736 10.1111/j.1365-294X.2005.02595.x

[16] Karakulak S, Oray I, Corriero A, Deflorio M, Santamaria N, et al (2004) Evidence of a spawning area for the bluefin tuna (*Thunnus thynnus* L.) in the eastern Mediterranean.. Journal Of Applied Ichthyology 20: 318–320.

[17] Hubisz MJ, Falush D, Stephens M, Pritchard JK (2009) Inferring weak population structure with the assistance of sample group information. Molecular Ecology Resources 9: 1322–1332. 21564903 10.1111/j.1755-0998.2009.02591.x PMC3518025

[18] Latch E, Dharmarajan G, Glaubitz JC, Rhodes OE J (2006) Relative performance of Bayesian clustering software for inferring population substructure and individual assignment at low levels of population differentiation. Conservation Genetics 7: 295–302.

[19] Francois O, Durand E (2010) Spatially explicit Bayesian clustering models in population genetics. Molecular Ecology Resources 10: 773–784. 21565089 10.1111/j.1755-0998.2010.02868.x

[20] Guillot G, Mortier F, Estoup A (2005) GENELAND: a computer package for landscape genetics. Molecular Ecology Notes 5: 712–715.

[21] Guillot G, Santos F, Estoup A (2008) Analysing georeferenced population genetics data with Geneland: a new algorithm to deal with null alleles and a friendly graphical user interface. Bioinformatics 24: 1406–1407. 18413327 10.1093/bioinformatics/btn136

[22] Cavalli-Sforza LL (1966) Population structure and human evolution. Proceedings of the Royal Society of London Series B-Biological Sciences 164: 362–379. 10.1098/rspb.1966.0038 4379525

[23] Johnson FM, Schaffer HE, Gillaspy JE, Rockwood ES (1969) Isozyme genotype-environment relationships in natural populations of the harvester ant, *Pogonomyrmex barbatus*, from Texas. Biochemical Genetics 3: 429–450. 4187937 10.1007/BF00485604

[24] Smouse PE, Spielman RS, Park MH (1982) Multiple-locus allocation of individuals to groups as a function of the genetic variation within and differences among human populations. American Naturalist 119: 445–463.

[25] Jombart T, Pontier D, Dufour AB (2009) Genetic markers in the playground of multivariate analysis. Heredity 102: 330–341. 19156164 10.1038/hdy.2008.130

[26] Goudet J (1995) Fstat version 1.2. A computer program to calculate F-statistics. Journal of Heredity 86: 485–486.

[27] Excoffier L (2005) Arlequin ver. 3.0: An integrated software package for population genetics data analysis. Evolutionary Bioinformatics Online 1: 47–50. PMC2658868 19325852

[28] Weir BS, Cockerham CC (1984) Estimating F-Statistics for the Analysis of Population-Structure. Evolution 38: 1358–1370. 28563791 10.1111/j.1558-5646.1984.tb05657.x

[29] Raymond M (1995) GENEPOP (version 1.2): population genetics software for exact tests and ecumenicism. Journal of Heredity 86: 248–249.

[30] Rice WR (1989) Analyzing Tables of Statistical Tests. Evolution 43: 223–225. 28568501 10.1111/j.1558-5646.1989.tb04220.x

[31] Pritchard JK, Stephens M, Donnelly P (2000) Inference of population structure using multilocus genotype data. Genetics 155: 945–959. 10835412 10.1093/genetics/155.2.945 PMC1461096

[32] Falush D, Stephens M, Pritchard JK (2003) Inference of population structure using multilocus genotype data: linked loci and correlated allele frequencies. Genetics 164: 1567–1587. 12930761 10.1093/genetics/164.4.1567 PMC1462648

[33] Evanno G, Regnaut S, Goudet J (2005) Detecting the number of clusters of individuals using the software STRUCTURE: a simulation study. Molecular Ecology 14: 2611–2620. 15969739 10.1111/j.1365-294X.2005.02553.x

[34] Jakobsson M, Rosenberg NA (2007) CLUMPP: a cluster matching and permutation program for dealing with label switching and multimodality in analysis of population structure. Bioinformatics 23: 1801–1806. 17485429 10.1093/bioinformatics/btm233

[35] Rosenberg N (2004) Distruct: a program for the graphical display of population structure. Molecular Ecology Notes 4: 137–138.

[36] R Development Core Team (2010) R: A Language and Environment for Statistical Computing. R Foundation for Statistical Computing, Vienna, Austria. URL http://www.R-project.org/.

[37] Manel S, Berthoud F, Bellemain E, Gaudeul M, Luikart G, et al (2007) A new individual-based spatial approach for identifying genetic discontinuities in natural populations. Molecular Ecology 16: 2031–2043. 17498230 10.1111/j.1365-294X.2007.03293.x

[38] Balding DJ (2003) Likelihood-based inference for genetic correlation coefficients. Theoretical population biology 63: 221–230. 12689793 10.1016/s0040-5809(03)00007-8

[39] Greenacre M (1966) Theory and Applications of Correspondence Analysis. Academic Press: London.

[40] Legendre P, Legendre L (1998) Numerical Ecology. 2nd English ed: Elsevier.

[41] Dray S, Dufour AB (2007) The ade4 package: implementing the duality diagram for ecologists. Journal of Statistical Software 22: 1–20.

[42] Jombart T (2008) adegenet: a R package for the multivariate analysis of genetic markers. Bioinformatics 24: 1403–1405. 18397895 10.1093/bioinformatics/btn129

[43] Oksanen J, Blanchet F, Kindt R, Legendre P, Minchin P, et al. (2013) vegan: Community Ecology Package. R package version 20–8: http://CRAN.R-project.org/package=vegan.

[44] Rousset F (1997) Genetic Differentiation and Estimation of Gene Flow from F-Statistics Under Isolation by Distance. Genetics 145: 1219–1228. 9093870 10.1093/genetics/145.4.1219 PMC1207888

[45] Mantel N (1967) The detection of disease clustering and a generalised regression approach. Cancer Research 27: 209–220. 6018555

[46] Smouse P, Long J, Sokal R (1986) Multiple regression and correlation extensions of the Mantel test of matrix correspondence. Systematic Zoology 35: 627–632.

[47] Alvarado BremerJR, Vinas J, Mejuto J, Ely B, Pla C (2005) Comparative phylogeography of Atlantic bluefin tuna and swordfish: the combined effects of vicariance, secondary contact, introgression, and population expansion on the regional phylogenies of two highly migratory pelagic fishes. Molecular Phylogenetics and Evolution 36: 169–187. 15904864 10.1016/j.ympev.2004.12.011

[48] Procaccini G, Ruggiero MV, Orsini L (2002) Genetic structure and distribution of microsatellite population genetic diversity in Posidonia oceanica in the Mediterranean basin. Bulletin of Marine Science 71: 1291–1297.

[49] Vinas J, Bremer JA, Pla C (2004) Phylogeography of the Atlantic bonito (*Sarda sarda*) in the northern Mediterranean: the combined effects of historical vicariance, population expansion, secondary invasion, and isolation by distance. Molecular Phylogenetics and Evolution 33: 32–42. 15324837 10.1016/j.ympev.2004.04.009

[50] Mejri R, Lo Brutto S, Ben Hassine OK, Arculeo M (2009) A study on *Pomatoschistus tortonesei* Miller 1968 (Perciformes, Gobiidae) reveals the Siculo-Tunisian Strait (STS) as a breakpoint to gene flow in the Mediterranean basin. Molecular Phylogenetics and Evolution 53: 596–601. 19422922 10.1016/j.ympev.2009.04.018

[51] Serra IA, Innocenti AM, Di Maida G, Calvo S, Migliaccio M, et al (2010) Genetic structure in the Mediterranean seagrass Posidonia oceanica: disentangling past vicariance events from contemporary patterns of gene flow. Molecular Ecology 19: 557–568. 20051010 10.1111/j.1365-294X.2009.04462.x

[52] Kaoueche M, Bahri-Sfar L, Gonzalez-Wanguemert M, Perez-Ruzafa A, Ben Hassine OK (2011) Allozyme and mtDNA variation of white seabream *Diplodus sargus* populations in a transition area between western and eastern Mediterranean basins (Siculo-Tunisian Strait). African Journal of Marine Science 33: 79–90.

[53] Reeb CA (2010) Genetic discontinuity of big fish in a small sea. Proceedings of the National Academy of Sciences of the United States of America 107: 2377–2378. 20139303 10.1073/pnas.0914639107 PMC2823861

[54] Block BA, Teo SLH, Walli A, Boustany A, Stokesbury MJW, et al (2005) Electronic tagging and population structure of Atlantic bluefin tuna. Nature 434: 1121–1127. 15858572 10.1038/nature03463

[55] De Metrio G, Arnold GP, Arnold GP, Block BA, Megalafonou P, et al (2005) Movements of bluefin tuna (*Thunnus thynnus* L.) tagged in the Mediterranean Sea with pop-up satellite tags. ICCAT Col Vol Sci Pap 58: 1337–1340.

[56] Sella M (1929) Migrazioni e habitat del tonno (*Thunnus thynnus* L.) studiati col metodo degli ami, con osservazioni su l’accrescimento, sul regime delle tonnare ecc. R Comitato Talassografico Italiano 16: 3–24.

[57] Sella M (1932) Il Tonno. Note dell’Istituto Italo-Germanico di Biologia Marina di Rovigno d’Istria. 3: 3–24.

[58] Fromentin JM, Powers JE (2005) Atlantic bluefin tuna: population dynamics, ecology, fisheries and management. Fish and Fisheries 6: 281–306.

[59] Rooker JR, Bremer JRA, Block BA, Dewar H, De Metrio G, et al (2007) Life history and stock structure of Atlantic bluefin tuna (*Thunnus thynnus*). Reviews in Fisheries Science 15: 265–310.

[60] Alemany F, Quintanilla L, Velez-Belchi P, Garcia A, Cortes D, et al (2010) Characterization of the spawning habitat of Atlantic bluefin tuna and related species in the Balearic Sea (western Mediterranean). Progress in Oceanography 86: 21–38.

[61] Muhling BA, Reglero P, Ciannelli L, Alvarez-Berastegui D, Alemany F, et al (2013) Comparison between environmental characteristics of larval bluefin tuna *Thunnus thynnus* habitat in the Gulf of Mexico and western Mediterranean Sea. Marine Ecology Progress Series 486: 257–276.

[62] Garcìa A, Cortés D, Quintanilla J, Ràmirez T, Quintanilla L, et al (2013) Climate-induced environmental conditions influencing interannual variability of Mediterranean bluefin (*Thunnus thynnus*) larval growth. Fisheries Oceanography 22: 273–287.

[63] Koched W, Hattour A, Alemany F, Garcìa A, Said K (2013) Spatial distribution of tuna larvae in the Gulf of Gabes (Eastern Mediterranean) in relation with environmental parameters. Mediterranean Marine Science 14: 5–14.

[64] Teo SLH, Boustany AM, Block BA (2007) Oceanographic preferences of Atlantic bluefin tuna, *Thunnus thynnus*, on their Gulf of Mexico breeding grounds. Marine Biology 152: 1105–1119.

[65] Mariani P, MacKenzie BR, Iudicone D, Bozec A (2010) Modelling retention and dispersion mechanisms of bluefin tuna eggs and larvae in the northwest Mediterranean Sea. Progress in Oceanography 86: 45–58.

[66] Reglero P, Ciannelli L, Alvarez-Berastegui D, Balbìn R, Lopez-Jurado JL, et al (2012) Geographically and environmentally driven spawning distributions of tuna species in the western Mediterranean Sea. Marine Ecology Progress Series 463: 273–284.

[67] Bergamasco A, Malanotte-Rizzoli P (2010) The circulation of the Mediterranean Sea: a historical review of experimental investigations. Advances in Oceanography and Limnology 1: 11–28.

[68] Poulain P-M, Menna M, Mauri E (2012) Surface geostrophic circulation of the Mediterranean Sea derived from drifter and satellite altimeter data. Journal of Physical Oceanography 42: 973–990.

[69] Galarza JA, Carreras-Carbonell J, Macpherson E, Pascual M, Roques S, et al (2009) The influence of oceanographic fronts and early-life-history traits on connectivity among littoral fish species. Proceedings of the National Academy of Sciences 106: 1473–1478. 10.1073/pnas.0806804106 PMC2629446 19164518

[70] Corander J, Waldmann P, Sillanpaa MJ (2003) Bayesian analysis of genetic differentiation between populations. Genetics 163: 367–374. 12586722 10.1093/genetics/163.1.367 PMC1462429

[71] Corander J, Marttinen P (2006) Bayesian identification of admixture events using multilocus molecular markers. Molecular Ecology 15: 2833–2843. 16911204 10.1111/j.1365-294X.2006.02994.x

[72] Francois O, Ancelet S, Guillot G (2006) Bayesian clustering using hidden Markov random fields in spatial population genetics. Genetics 174: 805–816. 16888334 10.1534/genetics.106.059923 PMC1602073

[73] Corander J, Siren J, Arjas E (2008) Bayesian spatial modeling of genetic population structure. Computational Statistics 23: 111–129.

