# Supplementary material for: Genetic Structure of Bluefin Tuna in the Mediterranean Sea Correlates with Environmental Variables
Source: PLoS One. 2013 Nov 18;8(11):e80105. doi: 10.1371/journal.pone.0080105 (PMC3832436; doi:10.1371/journal.pone.0080105)
Supplement: Table S1 — Sampling data of the Thunnus thynnus samples. (DOC) [file pone.0080105.s001.doc]

| Sample ID | Area | N | Age  classes* | Sampling year | Temperature (°C) | Salinity  (psu) | Latitude | Longitude |
| --- | --- | --- | --- | --- | --- | --- | --- | --- |
| **1) SAR** | Sardinian Traps | 29 | 5-18 | 2005 | 18.07 | 37.77 | 39.145 | 8.343 |
| **2) ADR** | Adriatic Sea | 73 | 5-17 | 2003-2005 | 16.72 | 38.07 | 42.826 | 14.844 |
| **3) LIG** | Ligurian Sea | 36 | 1-4 | 1999-2000 | 16.24 | 37.90 | 43.987 | 9.000 |
| **4) ALG** | Algerian coasts | 39 | 4-18 | 2006 | 19.10 | 36.88 | 37.053 | 2.619 |
| **5) ALB** | Alboran Sea | 40 | 7-11 | 2005 | 19.54 | 36.61 | 35.991 | -3.401 |
| **6) STY** | Tyrrhenian Sea | 39 | 4-24 | 2007 | 18.15 | 37.86 | 38.981 | 15.618 |
| **7) CYP** | Cyprus coasts | 60 | > 9 | 2008 | 19.74 | 39.23 | 34.670 | 33.59 |

*Age class was assessed based on Cort 1991 [7]
