# Supplementary material for: Genetic Structure of Bluefin Tuna in the Mediterranean Sea Correlates with Environmental Variables
Source: PLoS One. 2013 Nov 18;8(11):e80105. doi: 10.1371/journal.pone.0080105 (PMC3832436; doi:10.1371/journal.pone.0080105)
Supplement: Table S2 — Summary statistics at the seven microsatellite loci of Thunnus thynnus samples. (DOC) [file pone.0080105.s002.doc]

| **Sample** |  |  |  | **Locus** |  |  |  |
| --- | --- | --- | --- | --- | --- | --- | --- |
|  | **Tth5** | **Tth10** | **Tth34** | **Tth1-31** | **Tth208** | **Tth157** | **Tth62** |
| **ADR** |  |  |  |  |  |  |  |
| *n* | 71 | 73 | 73 | 73 | 71 | 73 | 71 |
| *a* | 3 | 5 | 17 | 18 | 26 | 7 | 13 |
| a*R* | 2.91 | 3.64 | 12.99 | 14.04 | 18.25 | 5.34 | 10.17 |
| a*S* | 123-131 | 112-136 | 103-183 | 90-128 | 140-206 | 117-129 | 83-113 |
| *H_E_* | 0.47 | 0.51 | 0.83 | 0.89 | 0.92 | 0.66 | 0.84 |
| *H_O_* | 0.42 | 0.55 | 0.73 | 0.84 | 0.70 | 0.51 | 0.79 |
| *HW* | 0.13 | 0.00* | 0.05 | 0.08 | 0.00* | 0.00* | 0.52 |
| **STY** |  |  |  |  |  |  |  |
| *n* | 39 | 39 | 39 | 39 | 38 | 38 | 39 |
| *a* | 3 | 2 | 12 | 17 | 19 | 6 | 12 |
| a*R* | 2.69 | 2 | 11.38 | 15.03 | 17.30 | 5.13 | 11.02 |
| a*S* | 123-131 | 116-120 | 107-187 | 92-140 | 140-206 | 117-127 | 83-115 |
| *H_E_* | 0.41 | 0.43 | 0.82 | 0.92 | 0.91 | 0.48 | 0.84 |
| *H_O_* | 0.46 | 0.56 | 0.92 | 0.92 | 0.87 | 0.58 | 0.85 |
| *HW* | 0.17 | 0.07 | 0.01 | 0.18 | 0.00* | 0.06 | 0.2 |
| **LIG** |  |  |  |  |  |  |  |
| *n* | 35 | 36 | 36 | 36 | 36 | 36 | 36 |
| *a* | 3 | 2 | 14 | 13 | 18 | 6 | 13 |
| a*R* | 3 | 2 | 12.48 | 12.19 | 16.34 | 5.75 | 11.69 |
| a*S* | 123-131 | 116-120 | 103-163 | 92-130 | 140-206 | 119-129 | 85-123 |
| *H_E_* | 0.54 | 0.48 | 0.84 | 0.90 | 0.92 | 0.70 | 0.87 |
| *H_O_* | 0.43 | 0.44 | 0.86 | 0.86 | 0.69 | 0.61 | 0.78 |
| *HW* | 0.11 | 0.73 | 0.46 | 0.41 | 0.01 | 0.03 | 0.03 |
| **SAR** |  |  |  |  |  |  |  |
| *n* | 28 | 29 | 29 | 29 | 29 | 29 | 28 |
| *a* | 3 | 2 | 11 | 10 | 17 | 4 | 10 |
| a*R* | 3 | 2 | 10.85 | 9.86 | 16.64 | 4 | 9.89 |
| a*S* | 123-131 | 116-120 | 103-147 | 92-126 | 140-208 | 117-125 | 83-109 |
| *H_E_* | 0.49 | 0.41 | 0.81 | 0.83 | 0.92 | 0.45 | 0.85 |
| *H_O_* | 0.32 | 0.34 | 0.79 | 0.72 | 0.76 | 0.41 | 0.82 |
| *HW* | 0.09 | 0.64 | 0.26 | 0.77 | 0.02 | 0.28 | 0.04 |
| **ALG** |  |  |  |  |  |  |  |
| *n* | 39 | 39 | 38 | 38 | 39 | 39 | 39 |
| *a* | 3 | 2 | 17 | 15 | 18 | 6 | 11 |
| a*R* | 3 | 2 | 14.07 | 12.87 | 15.96 | 5.79 | 10.26 |
| a*S* | 123-131 | 116-120 | 103-187 | 90-132 | 140-216 | 119-129 | 83-115 |
| *H_E_* | 0.53 | 0.49 | 0.75 | 0.87 | 0.91 | 0.62 | 0.82 |
| *H_O_* | 0.38 | 0.54 | 0.74 | 0.84 | 0.69 | 0.62 | 0.90 |
| *HW* | 0.07 | 0.74 | 0.90 | 0.07 | 0.00* | 0.87 | 0.72 |
| **ALB** |  |  |  |  |  |  |  |
| *n* | 40 | 40 | 40 | 40 | 38 | 40 | 40 |
| *a* | 3 | 2 | 13 | 13 | 18 | 4 | 10 |
| a*R* | 2.99 | 2 | 11.87 | 11.81 | 16.55 | 4 | 8.81 |
| a*S* | 123-131 | 116-120 | 103-155 | 92-138 | 140-194 | 119-125 | 83-111 |
| *H_E_* | 0.49 | 0.51 | 0.83 | 0.90 | 0.93 | 0.50 | 0.74 |
| *H_O_* | 0.60 | 0.57 | 0.80 | 0.80 | 0.87 | 0.40 | 0.65 |
| *HW* | 0.01 | 0.52 | 0.97 | 0.01 | 0.23 | 0.01 | 0.00* |
| **CYP** |  |  |  |  |  |  |  |
| *n* | 60 | 60 | 60 | 60 | 60 | 59 | 58 |
| *a* | 3 | 3 | 15 | 14 | 20 | 5 | 17 |
| a*R* | 2.97 | 2.45 | 11.82 | 12.11 | 14.99 | 4.87 | 13.69 |
| a*S* | 123-131 | 104-120 | 107-187 | 92-130 | 140-194 | 119-127 | 75-117 |
| *H_E_* | 0.44 | 0.49 | 0.78 | 0.89 | 0.89 | 0.57 | 0.87 |
| *H_O_* | 0.41 | 0.55 | 0.68 | 0.88 | 0.78 | 0.64 | 0.86 |
| *HW* | 0.13 | 0.76 | 0.31 | 0.32 | 0.004* | 0.22 | 0.76 |

*n*, number of individuals; *a*, number of alleles; *aR*, allelic richness per locus and sample; *aS*, allele size range; *H_E_*, expected heterozygosity; *H_O_*, observed heterozygosity; *HW*, Hardy-Weinberg equilibrium test. * significant loci after sequential Bonferroni correction.
