# Supplementary material for: Genetic Structure of Bluefin Tuna in the Mediterranean Sea Correlates with Environmental Variables
Source: PLoS One. 2013 Nov 18;8(11):e80105. doi: 10.1371/journal.pone.0080105 (PMC3832436; doi:10.1371/journal.pone.0080105)
Supplement: Table S3 — Gene diversity and Hardy-Weinberg equilibrium deviation test of Thunnus thynnus samples. (DOC) [file pone.0080105.s003.doc]

| Sample | N | Gene Diversity | | | P_HW_ |
| --- | --- | --- | --- | --- | --- |
|  |  | ã*R* | H_e_ | H_o_ |  |
| ADR | 73 | 9.7 | 0.73 | 0.64 | 0.0000* |
| STY | 39 | 9.2 | 0.69 | 0.74 | NS |
| LIG | 36 | 9.0 | 0.75 | 0.67 | 0.0000* |
| SAR | 29 | 8.0 | 0.68 | 0.60 | 0.0003* |
| ALG | 39 | 9.1 | 0.71 | 0.67 | 0.0008* |
| ALB | 40 | 8.3 | 0.70 | 0.67 | 0.0053* |
| CYP | 60 | 9.0 | 0.71 | 0.69 | 0.0402* |

N is the sample size; ã*R* is the mean allelic richness; *H_e_* is the mean expected heterozygosity; *H_o_*, is the mean observed heterozygosity); P_HW_ is the P-value of the Hardy-Weinberg equilibrium deviation test; *significant after sequential Bonferroni correction.
