# Supplementary material for: Genetic Structure of Bluefin Tuna in the Mediterranean Sea Correlates with Environmental Variables
Source: PLoS One. 2013 Nov 18;8(11):e80105. doi: 10.1371/journal.pone.0080105 (PMC3832436; doi:10.1371/journal.pone.0080105)
Supplement: Table S4 — Percentages of variations explained by CA and CCA axis. (DOC) [file pone.0080105.s004.doc]

| Analysis | First Axis | Second Axis | Third Axis |
| --- | --- | --- | --- |
| CA | 33% | 17% | 16% |
| CCA | 72% | 27% |  |
