# Supplementary material for: Genetic Structure of Bluefin Tuna in the Mediterranean Sea Correlates with Environmental Variables
Source: PLoS One. 2013 Nov 18;8(11):e80105. doi: 10.1371/journal.pone.0080105 (PMC3832436; doi:10.1371/journal.pone.0080105)
Supplement: Table S5 — Ordination scores of mean temperature (Mean-t) and mean salinity (Mean-S) values. (DOC) [file pone.0080105.s005.doc]

| **CCA scores** |  |  |
| --- | --- | --- |
| Variable | First Axis | Second Axis |
| Mean-t | 0.999 | -0.0344 |
| Mean-S | -0.0454 | -0.998 |
