# Supplementary material for: Genetic Structure of Bluefin Tuna in the Mediterranean Sea Correlates with Environmental Variables
Source: PLoS One. 2013 Nov 18;8(11):e80105. doi: 10.1371/journal.pone.0080105 (PMC3832436; doi:10.1371/journal.pone.0080105)
Supplement: Text S1 — DNA extraction, Microsatellite marker analysis protocols, Correspondence Analysis (CA) and Canonical/Constrained Correspondence Analysis (CCA) results and references. (DOC) [file pone.0080105.s006.doc]

**Protocols**

**DNA extraction.** Total genomic DNA from ABFTs collected from Cyprus coasts was extracted from ethanol stored specimens of soft tissues (fin, white skeletal muscle, gill) according to a standard CTAB-protocol [1].

**Microsatellite marker analysis.** Seven microsatellite loci (*Tth*5, *Tth*10, *Tth*34 [2]; *Tth*1-31, *Tth*208, *Tth*157, *Tth*62 [3]) were selected as markers for genetic analysis.

Particular care was taken in avoiding technical artefacts potentially leading to erroneous genotyping, including the effects of contamination, allele dropout and detection of false alleles. To rule out contamination by exogenous DNA, we had negative controls for all DNA extraction and amplification steps. The accuracy of allele scoring was tested by sequencing PCR products at all loci in a subset of 20 individuals. Microsatellite loci were amplified by using the following PCR conditions: 3 min denaturation at 94°C, followed by 35 cycles 30 sec at 94°C, 30 sec at annealing temperature and 30 sec at 72°C for extension. An extra final extension of 3 min 72°C was added after the last cycle. Amplifications of DNA were carried out in 10 μl reaction 10 mM Tris-HCl (pH 9), 50 mM KCl, 0.1% Triton X-100, 1.5 mM MgCl_2_, 0.2 mM of each dNTP, 0.5 μM of each primer, 0.5 U *Taq* DNA Polymerase (Promega) and 20 ng genomic DNA. To genotype individuals, we assessed the allele size on a ABIPrism 310 automatic sequencer (Applied Biosystems) using the labelled forward primers (6-FAM, HEX, or TAMRA; MWG Biotech) and the ROX 500 (Applied Biosystem) as internal standard. Allele sizing was determined by analyzing PCR products using the Software GeneScan Analysis v. 2.02 (Applied Biosystem).

Results

|  |
| --- |

**Correspondence Analysis (CA)**

Correspondence analysis is a method of ‘ordination in reduced space’ and therefore its purpose is to summarize a strongly multivariate dataset into a small set of uncorrelated synthetic variables. It is similar to principal component analysis, but instead of preserving Euclidean distance among observations, χ^2^ distance is preserved. CA is appropriate for the analysis of a contingency table, that is, a matrix of positive integers [4], and is thus appropriate for the analysis of a table of allele counts. This method decomposes the χ^2^ statistic associated to the allele counts contingency table into orthogonal factors that can be used to draw maps of points representing variables or their categories.

**Canonical/constrained Correspondence Analysis (CCA)**

The CCA is a direct gradient analysis method that concomitantly analyses the genetic and environmental data. It is the canonical form of correspondence analysis, any data table that could be subjected to correspondence analysis forms a suitable response matrix for CCA. χ^2^ transformed data matrix is subjected to weighted linear regression on constraining variables, and the fitted values are submitted to correspondence analysis performed via singular value decomposition. CCA involves

computation of the matrix of covariances between explanatory variables [5] therefore requires that the number of explanatory variables (for instance, environmental variables) be fairly lower than the number of studied objects (genotypes or populations) to be computable. Canonical correspondence analysis is a constrained method: CCA does not try to display all variation in the data, but only the part that can be explained by the used constraints.

CCA [6] have proven useful in population genetics, mostly to investigate the portion of the genetic variability that can be explained by a set of environmental variables. This multivariate method indeed constructs linear combinations of environmental variables along which the population samples are maximally separated.

|  |  |  |  |  |  |  |  |  |
| --- | --- | --- | --- | --- | --- | --- | --- | --- |
|  |  |  |  |  |  |  |  |  |
|  |  |  |  |  |  |  |  |  |
|  |  |  |  |  |  |  |  |  |
|  |  |  |  |  |  |  |  |  |
|  |  |  |  |  |  |  |  |  |
|  |  |  |  |  |  |  |  |  |
|  |  |  |  |  |  |  |  |  |

|  |  |  |  |  |  |  |  |
| --- | --- | --- | --- | --- | --- | --- | --- |
|  |  |  |  |  |  |  |  |
|  |  |  |  |  |  |  |  |
|  |  |  |  |  |  |  |  |
|  |  |  |  |  |  |  |  |
|  |  |  |  |  |  |  |  |
|  |  |  |  |  |  |  |  |
|  |  |  |  |  |  |  |  |
|  |  |  |  |  |  |  |  |
|  |  |  |  |  |  |  |  |
|  |  |  |  |  |  |  |  |
|  |  |  |  |  |  |  |  |
|  |  |  |  |  |  |  |  |
|  |  |  |  |  |  |  |  |
|  |  |  |  |  |  |  |  |
|  |  |  |  |  |  |  |  |
|  |  |  |  |  |  |  |  |
|  |  |  |  |  |  |  |  |
|  |  |  |  |  |  |  |  |
|  |  |  |  |  |  |  |  |
|  |  |  |  |  |  |  |  |
|  |  |  |  |  |  |  |  |
|  |  |  |  |  |  |  |  |
|  |  |  |  |  |  |  |  |
|  |  |  |  |  |  |  |  |
|  |  |  |  |  |  |  |  |
|  |  |  |  |  |  |  |  |
|  |  |  |  |  |  |  |  |
|  |  |  |  |  |  |  |  |
|  |  |  |  |  |  |  |  |
|  |  |  |  |  |  |  |  |
|  |  |  |  |  |  |  |  |
|  |  |  |  |  |  |  |  |
|  |  |  |  |  |  |  |  |
|  |  |  |  |  |  |  |  |
|  |  |  |  |  |  |  |  |
|  |  |  |  |  |  |  |  |
|  |  |  |  |  |  |  |  |
|  |  |  |  |  |  |  |  |
|  |  |  |  |  |  |  |  |
|  |  |  |  |  |  |  |  |
|  |  |  |  |  |  |  |  |
|  |  |  |  |  |  |  |  |
|  |  |  |  |  |  |  |  |
|  |  |  |  |  |  |  |  |
|  |  |  |  |  |  |  |  |
|  |  |  |  |  |  |  |  |
|  |  |  |  |  |  |  |  |
|  |  |  |  |  |  |  |  |
|  |  |  |  |  |  |  |  |
|  |  |  |  |  |  |  |  |
|  |  |  |  |  |  |  |  |
|  |  |  |  |  |  |  |  |
|  |  |  |  |  |  |  |  |
|  |  |  |  |  |  |  |  |
|  |  |  |  |  |  |  |  |
|  |  |  |  |  |  |  |  |
|  |  |  |  |  |  |  |  |

|  |  |  | | |  |
| --- | --- | --- | --- | --- | --- |
|  |  |  |  |  |  |
|  |  |  |  |  |  |
|  |  |  |  |  |  |
|  |  |  |  |  |  |
|  |  |  |  |  |  |
|  |  |  |  |  |  |
|  |  |  |  |  |  |
|  |  |  |  |  |  |

|  |  |  |  |
| --- | --- | --- | --- |
|  |  |  |  |
|  |  |  |  |

|  |  |  |
| --- | --- | --- |
|  |  |  |
|  |  |  |
|  |  |  |

**References**

1. Winnepenninckx B., Backeljau T., De Wachter R. 1993 Extraction of high molecular weight DNA from mollusks. *Trends in Genetics* **9**, 407.

2. McDowell J.R., Diaz-Jaimes P., Graves J.E. 2002 Isolation and characterization of seven tetranucleotide microsatellite loci from Atlantic northern bluefin tuna Thunnus thynnus thynnus. *Molecular Ecology Notes* **2**(3), 214-216. (doi:10.1046/j.1471-8286.2002.00197.x).

3. Clark T.B., Ma L., Saillant E., Gold J.R. 2004 Microsatellite DNA markers for population-genetic studies of Atlantic bluefin tuna (Thunnus thynnus thynnus) and other species of genus Thunnus. *Molecular Ecology Notes* **4**(1), 70-73.

4. Greenacre M. 1966 Theory and Applications of Correspondence Analysis. *Academic Press: London*.

5. Legendre P., Legendre L. 1998 Numerical Ecology. *2nd English ed*, Elsevier.

6. Angers B., Plante M., Bernatchez L. 1999 Canonical correspondence analysis for estimating spatial and environmental effects on microsatellite gene diversity in brook charr (*Salvelinus fontinalis*). *Molecular Ecology* **8**, 1043–1053.

7. Cort, J.L. 1991. Age and Growth of the Bluefin Tuna, *Thunnus thynnus* (L.) of the Northwest Atlantic. ICCAT. SCRS/1990/66. *Collective Volume of Scientific Papers, ICCAT* **35**(2), 213-230.
